# Trends in Positive Urine Culture Rates and Antimicrobial Resistance in Non-Hospitalized Children from Western Romania: A Retrospective Observational Study

**DOI:** 10.3390/antibiotics14070723

**Published:** 2025-07-18

**Authors:** Constantin Catalin Marc, Maria Daniela Mot, Monica Licker, Delia Muntean, Daniela Teodora Marti, Ana Alexandra Ardelean, Alina Ciceu, Sergiu Adrian Sprintar, Daniela Adriana Oatis, Alin Gabriel Mihu, Tudor Rares Olariu

**Affiliations:** 1 Department of General Medicine, Doctoral School, Victor Babes University of Medicine and Pharmacy, 300041 Timisoara, Romania; catalin.marc@umft.ro; 2 Department of Biology and Life Sciences, Faculty of Medicine, Vasile Goldis Western University of Arad, 310025 Arad, Romania; mot.dana@uvvg.ro (M.D.M.); dana_m73@yahoo.com (D.T.M.); 3 Microbiology Department, Multidisciplinary Research Center of Antimicrobial Resistance, Victor Babes University of Medicine and Pharmacy, 300041 Timisoara, Romania; licker.monica@umft.ro (M.L.); muntean.delia@umft.ro (D.M.); 4 Microbiology Laboratory, “Pius Brinzeu” County Clinical Emergency Hospital, 300723 Timisoara, Romania; 5 Discipline of Parasitology, Department of Infectious Diseases, Victor Babes University of Medicine and Pharmacy, 300041 Timisoara, Romania; paduraru.ana@umft.ro (A.A.A.); rolariu@umft.ro (T.R.O.); 6 Center for Diagnosis and Study of Parasitic Diseases, Department of Infectious Disease, Victor Babes University of Medicine and Pharmacy, 300041 Timisoara, Romania; sergiu.sprintar@umft.ro; 7 Patogen Preventia, 300124 Timisoara, Romania; 8 Clinical Laboratory, Municipal Clinical Emergency Hospital, 300254 Timisoara, Romania; 9 “Aurel Ardelean” Institute of Life Sciences, Vasile Goldis Western University of Arad, 86 Rebreanu, 310414 Arad, Romania; ciceu.alina@uvvg.ro; 10 Bioclinica Medical Analysis Laboratory, Dreptatii Street, nr. 23, 310300 Arad, Romania

**Keywords:** children, Romania, urinary tract infection, UTI, *E. coli*, *Escherichia coli*, antibiotic resistance

## Abstract

Background: Urinary tract infections (UTIs) are among the most common types of infections during childhood. Limited data are available on the prevalence of UTI in children from Romania, with most being available for hospitalized children. For this reason, we conducted a retrospective observational study in consecutive non-hospitalized children to assess the number of positive UTI samples and the antibacterial resistance of causative pathogens. Methods: This study included 7222 consecutive urine cultures collected from children aged 1 to 18 years who are residents of Arad County, Western Romania. Urine samples were analyzed for leukocyturia and cultures for the presence of monomorphic bacteria. Results: The overall number of positive UTI samples was 10.44%. A higher number of positive UTI samples was observed in females when compared to males and in children aged 6–12 and 12 to 18 years when compared to those aged 1–5 years. The antibiotic susceptibility testing of *E. coli* isolates revealed high sensitivity to most tested antibacterials. Near-complete susceptibility was observed for fosfomycin (99.71%) and nitrofurantoin (96.01%), while high susceptibility rates were also observed for ciprofloxacin (85.43%) and amoxicillin–clavulanic acid (75.05%). In contrast, high resistance was found for ampicillin (62.28% resistant) and trimethoprim–sulfamethoxazole (36.53% resistant). Conclusions: Given the clinical risks associated with UTI in children, our findings underscore the urgent need for the continued monitoring of multidrug-resistant strains. Our study provides important epidemiological and resistance data to guide empirical treatment and strengthen pediatric antimicrobial resistance surveillance. Future studies should focus on different regions and regularly update resistance patterns to keep treatment and prevention strategies aligned with local conditions.

## 1. Introduction

Urinary tract infections (UTIs) are among the most common bacterial infections worldwide, encompassing a spectrum of syndromes mediated as an inflammatory response against pathogenic bacteria and affecting the urinary tract. UTIs occur in both community and healthcare settings [1,2,3]. The UTI population represents a serious medical concern due to the potential for both immediate and long-term health problems [4]. Short-term morbidity can arise from infection within the renal system, while long-term morbidity follows renal injury and scarring from upper tract UTIs [5].

UTIs in children refer to the presence of pathogenic microorganisms within the urinary system, including the kidneys, ureters, bladder, or urethra [6,7]. Approximately one in ten children may experience a UTI [8]. The prevalence is about 1.5 times higher in girls than in boys, and approximately one in seven UTIs are caused by extended-spectrum beta-lactamase (ESBL)-producing *Enterobacteriaceae* [9]. Children with vesicoureteral reflux, a history of previous UTI, or recent antibiotic use are considered at high risk, and infections caused by ESBL-producing pathogens are associated with prolonged hospital stays [8,9]. UTIs are particularly concerning in the pediatric population because they can rapidly progress to pyelonephritis, which may lead to kidney damage [10].

The signs and symptoms of a UTI in children may vary depending on the child`s age. In younger children, UTIs often present with non-specific signs, such as fever, poor feeding, and vomiting, while older children typically exhibit symptoms like dysuria, abdominal pain, frequent urination, and flank tenderness [6,10].

UTIs in children were reported to be predominantly caused by *Escherichia coli*, which remains the most prevalent uropathogen in this population [11]. Because of its capacity to adhere and infiltrate the lining of the urinary system, uropathogenic *E. coli* is especially harmful when it comes to UTIs in children because of its multiple virulence factors, which include cytotoxic necrotizing factor 1 and pyelonephritis-associated pili [12].

Currently, there is limited data regarding the prevalence of UTIs in Romania, and the studies published so far mostly reported the prevalence of UTIs among hospitalized children [13,14,15]. To address this gap, we conducted a five-year retrospective observational study to assess the number of positive UTI samples from non-hospitalized children and to identify the primary pathogens causing this medical condition. In addition, we investigated the antibacterial resistance patterns of *E. coli*, *Proteus mirabilis*, *Klebsiella pneumoniae*, and *Enterococcus faecalis*, the most common pathogens identified in urine cultures.

## 2. Results

Samples collected from 7222 children were included in the study. The mean age of participants was 6.65 ± 4.85 years. Of the 7222 participants, 2548 (35.28%) were males, and 5555 (76.92%) were residents of urban areas. The overall number of positive UTI samples was 10.44% (754 of 7222 children) (Table 1).

Children aged 6–12 years had a significantly higher number of positive UTI samples (11.99%) compared to those aged 1–5 years (8.88%) (cOR = 1.4; 95% CI: 1.18–1.65; *p* < 0.001). Similarly, adolescents aged 13–18 years showed a positive UTI sample number of 12.5%, with 47% higher odds of infection compared to the youngest age group (cOR = 1.47; 95% CI: 1.19–1.81; *p* < 0.001).

Gender-stratified analysis revealed that females had a markedly higher number of positive UTI samples (13.04%) compared to males (5.63%), with females showing 2.51 times higher odds of UTI (cOR = 2.51; 95% CI: 2.08–3.03; *p* < 0.001).

No statistically significant difference in the number of positive UTI samples was found between children living in rural areas (10.86%) and those from urban settings (10.32%) (cOR = 0.94; 95% CI: 0.79–1.13; *p* = 0.53) (Table 1).

The yearly number of positive UTI samples ranged from 9.88% to 12.41%, with the highest rate in 2016 (12.41%); this was used as the reference year in the univariate logistic regression model. The lowest prevalence was observed in 2022 (9.88%), corresponding to a crude odds ratio of 0.77 (95% CI: 0.58–1.03; *p* = 0.08) (Table 2).

Multivariate logistic regression analysis revealed several significant associations with UTI presence in the pediatric population (Table 3). Children aged 6–12 years and adolescents aged 13–18 years had significantly higher odds of presenting a positive UTI sample compared to those aged 1–5 years (used as reference group), indicating an increased risk with advancing age. Gender was strongly associated with UTI, with an adjusted odds ratio of 2.45 (95% CI: 2.03–2.97; *p* < 0.001), indicating that females had more than twice the risk of presenting a positive UTI sample compared to males. The area of residence (urban vs. rural) was not significantly associated with UTI risk (aOR = 0.98; 95% CI: 0.82–1.17; *p* = 0.81) (Table 3).

Among the 754 UTI samples, *E. coli* was the most frequently isolated pathogen, accounting for 66.45% of cases. *Proteus mirabilis* followed at 12.6%, with other notable isolates including *Klebsiella pneumoniae* (5.31%), *Enterococcus faecalis* (4.64%), and *Pseudomonas aeruginosa* (2.52%). Less common pathogens included *Staphylococcus saprophyticus* and *Streptococcus agalactiae* (each 1.19%) (Table 4).

In our study, the antibacterial susceptibility profile of *E. coli* isolates revealed high sensitivity to the majority of tested agents. Complete susceptibility (100%) was recorded for all carbapenems—ertapenem, imipenem, and meropenem—and near-complete susceptibility was recorded for fosfomycin (99.71%) and nitrofurantoin (96.01%). High sensitivity rates were also observed for gentamicin (90.62%), ciprofloxacin (85.43%), and third- and fourth-generation cephalosporins, including cefotaxime (88.08%), ceftazidime (87.23%), and cefepime (89.36%). Combination therapies such as amoxicillin + clavulanic acid (75.05%) and piperacillin + tazobactam (86.97%) demonstrated similarly favorable profiles. In contrast, the lowest susceptibility was noted for ampicillin, with only 36.93% of isolates being sensitive and 62.28% being resistant. Moderate resistance rates were also observed for norfloxacin (44.61%) and trimethoprim + sulfamethoxazole (36.53%). Overall, the isolates demonstrated a high degree of susceptibility to most antibiotics, particularly carbapenems and fosfomycin (Table 5).

Multinomial logistic regression confirmed year-to-year gains in *E. coli* susceptibility for almost every antibiotic tested between 2016 and 2022. Among β-lactams, ampicillin climbed from 30% to 42% susceptible (RRR 0.90, 95% CI 0.82–0.99); amoxicillin–clavulanate moved from the low 70% range to roughly 81% by 2021–2022 (first significant rise in 2018, RRR 0.80, 95% CI 0.68–0.93); piperacillin–tazobactam maintained ≥83% activity yet still improved significantly in 2018 (RRR 0.69, 95% CI 0.55–0.88). Third- and fourth-generation cephalosporins also rebounded: Cefotaxime surged from 78% to 94% and cefepime from 82% to 91%, each registering highly significant increases in 2018 (RRR 0.40 and 0.67, respectively), whereas ceftazidime showed a more modest but significant rise (RRR 0.74, 95% CI 0.60–0.90). The most dramatic change was seen with amikacin, for which its susceptibility leapt from 52% to 96% (RRR 0.49, 95% CI 0.40–0.59). Fluoroquinolone behavior diverged: Ciprofloxacin remained stably high (80–92% susceptible, only a transient 2018 uptick), but norfloxacin plummeted from >80% susceptibility before 2019 to 0% by 2021–2022 (early signal in 2018, RRR 2.29, 95% CI 1.94–2.71). Finally, trimethoprim–sulfamethoxazole showed a small yet significant improvement in 2018 (RRR 0.90, 95% CI 0.82–0.99), increasing overall susceptibility from 25% to 35% (Table 6).

*Proteus mirabilis* isolates presented a generally favorable susceptibility profile to several key antibiotics. The complete susceptibility (100%) of *Proteus mirabilis* isolates was observed for meropenem, while near-complete susceptibility rates were recorded for ertapenem (96.3%) and amikacin (89.41%). High levels of susceptibility were also noted for third- and fourth-generation cephalosporins, including cefotaxime (88.5%), ceftazidime (86.32%), and cefepime (85.88%), as well as for ciprofloxacin (85.27%) and gentamicin (84.21%). Combination agents such as piperacillin + tazobactam (87.36%) and amoxicillin + clavulanic acid (78.21%) maintained strong activity. In contrast, nitrofurantoin demonstrated complete resistance (100%), and imipenem showed low full susceptibility (23.16%) with a high rate of intermediate responses (48.42%). Moderate to high resistance was also observed for trimethoprim + sulfamethoxazole (54.74%) and norfloxacin (37.31%) (Table 7).

*Klebsiella pneumoniae* isolates presented high susceptibility to several antibiotics, particularly carbapenems and aminoglycosides. Complete susceptibility (100%) was observed for both ertapenem and gentamicin, while near-complete susceptibility was seen with imipenem (95%), meropenem (95%), and amikacin (94.87%). Third- and fourth-generation cephalosporins also demonstrated strong activity, with susceptibility rates of 74.36% for both cefotaxime and cefepime and 72.5% for ceftazidime. Among combination agents, amoxicillin + clavulanic acid (62.5%) and piperacillin + tazobactam (62.5%) showed moderate effectiveness. All isolates were resistant to ampicillin (100%), and notable resistance rates were found for ciprofloxacin (40%), norfloxacin (56.52%), fosfomycin (55%), and trimethoprim + sulfamethoxazole (37.5%). Nitrofurantoin demonstrated limited activity with only 25.71% susceptibility (Table 8).

*Enterococcus faecalis* isolates displayed overall high susceptibility, with several first-line agents retaining full or near-full activity. Ampicillin and tigecycline were universally effective, each showing 100% susceptibility. Glycopeptides and oxazolidinones also performed strongly: Vancomycin and teicoplanin each achieved 94.29% susceptibility, while linezolid reached 94.12%. Fluoroquinolone coverage remained high, as 85.71% of isolates were susceptible to ciprofloxacin. Aminoglycoside synergy testing showed mixed results: High-level gentamicin retained 80% susceptibility, whereas high-level streptomycin dropped to 62.86%, with resistance rates of 20% and 37.14%, respectively. Imipenem only exhibited moderate activity, with 58.82% fully susceptible and the remaining 41.18% falling into the intermediate category; no outright resistance was detected (Table 9).

## 3. Discussion

In this study, we reported a 10.44% rate of positive UTI samples in a large research sample of non-hospitalized children from Western Romania. These findings were higher than the 5.9% prevalence reported in a study conducted by O’Brien et al. (2013) in the United Kingdom, based on data from 1003 children in general practices between 2008 and 2010 [17]. Our finding was similar to the prevalence of 11.1% reported by Daniel et al. (2023) in a sample of 10,006 urine cultures from outpatient children in Warsaw, Poland [18], but they were lower than the 25.8% prevalence found in a study by Alrasheedy et al. (2021), which focused on 1083 children selected from tertiary hospitals in Saudi Arabia [19]. The observed variation in UTI prevalence across these studies can be attributed to several factors, including the child’s health status, access to healthcare, socioeconomic conditions, and cultural practices [17,19,20]. In regions with more advanced healthcare systems, increased access to medical care and diagnostic testing tends to result in higher rates of diagnosis and, consequently, a higher reported prevalence of UTIs. Socioeconomic status and hygiene practices are also critical determinants; areas with lower sanitation standards or limited access to clean water tend to exhibit higher UTI rates. Additionally, cultural factors, such as toilet training practices and parental attitudes toward hygiene, have been shown to influence UTI prevalence in children [20,21,22,23].

In the present study, the number of positive UTIs increased with age from 8.88% (164/1850) in children aged 1–5 years to 11.99% (132/1100) in those aged 6–12 years and 12.5% (50/400) in adolescents aged 13–18 years. This trend contrasts with the findings of Daniel et al. (2023), who reported the highest concentration of UTI cases (64.4%) in younger children, with a marked decline in incidence among older children, particularly after the age of seven [18]. Similarly, Boon et al. (2022) also observed a higher prevalence of UTIs in younger children [24]. A potential contributing factor to the high prevalence of *E. coli* in pediatric UTIs, especially among older age groups, may be dietary exposure to contaminated chicken meat. Studies have shown that chicken meat may be frequently contaminated with *E. coli*, including multidrug-resistant strains, as demonstrated in investigations conducted in the United States [25] and Canada [26]. Similarly, a study conducted in Romania identified multidrug-resistant *E. coli* strains in chicken meat [27], which is among the most widely consumed types of meat globally [25,26,28]. Members of the *Enterobacteriaceae* family have been shown to cause cross-contamination in domestic kitchens during chicken preparation, even when standard hygiene measures were followed [29,30], which may increase the risk of infection in children through indirect exposure within the household environment.

Our results indicate a significantly higher number of positive UTI samples in females compared to males, consistent with the findings of Miron et al. (2021) [14] and Isac et al. (2024) [31]. This difference was primarily attributed to anatomical differences; females have a shorter urethra and a urethral opening closer to the anus, which facilitates the ascent of bacteria to the bladder [32]. Additionally, the fecal–perineal–urethral hypothesis supports the notion that bacteria from the anus, especially *E. coli*, can migrate to the urethra, leading to infection. Studies demonstrated that *E. coli* strains from the rectal flora often dominate UTIs, supporting this pathway for bacterial transmission [33]. During puberty, hormonal changes (particularly the rise in estrogen) play a crucial role in shaping the vaginal environment. Estrogen promotes the maturation of vaginal tissues and stimulates glycogen production, which in turn supports the growth of *Lactobacillus* species that convert glycogen into lactic acid. This process lowers the vaginal pH, creating an acidic environment that protects against harmful bacteria [34]. In prepubertal girls, it is believed that lower estrogen levels and a higher vaginal pH may make them more vulnerable to regional infections [35]. As estrogen levels increase during puberty, the vaginal microbiota becomes dominated by *Lactobacillus* species, providing greater protection and reducing the risk of infections [5,34,36].

We reported no statistically significant difference in the number of positive UTI samples among children living in urban versus rural environments. A study conducted in Taiwan by Cheng et al. (2016) between 2009 and 2012 focused on ESBL *E. coli* and highlighted the same finding [37]. Previous research showed that service adaptations implemented during and following major medical crises, such as the COVID-19 pandemic, may reduce existing disparities, even though rural healthcare provision was often thought to be limited by factors including staffing shortages and hospital distance. The absence of statistical difference in UTI prevalence between urban and rural children in our study may result from enhanced care access together with improved service communication and hybrid healthcare delivery models that integrate online and in-person services [38,39].

We found a slight decrease in the number of positive UTI samples in children during the COVID-19 pandemic, although this was not statistically significant. These results align with a retrospective observational cohort study conducted in the United States that highlighted a decrease in UTI diagnoses during the early stages of the pandemic, without statistically significant increases in the short-term indicators of UTI severity. Several factors may have contributed to this decline in reported UTI cases, such as reduced healthcare utilization and changes in health-seeking behavior [40,41,42].

We identified *E. coli* as the most common pathogen causing UTIs in children from our region, similarly to other reports from Romania for both children and adults [14,15]. These findings are consistent with Werbel at al. (2021), who reported *E. coli* to be responsible for up to 80–90% of all pediatric UTIs [43]. Similar findings were reported by several studies throughout the world [44,45,46,47,48]. Factors such as anatomical anomalies in the urinary tract and genetic susceptibility contribute to this higher prevalence [43,49]. In terms of age distribution, *E. coli* is the dominant pathogen across various pediatric age groups, although its relative frequency may vary with age and gender [50,51].

In addition to *E. coli*, other pathogens such as *Klebsiella*, *Proteus*, and *Enterococcus* species have been reported, although they are less common than *E. coli*. Kılıç and Küçükkelepçe observed that *E. coli* was responsible for 61.2% of pediatric UTI cases in Turkey, followed by *Klebsiella pneumoniae* (13.3%) and *Proteus mirabilis* (9.1%) [52]. Given the consistent findings of *E. coli* as the predominant uropathogen across different geographical locations, it is crucial to incorporate regional data into clinical decision-making and antibiotic treatment guidelines to ensure effective empirical therapy [43,51].

We reported the complete susceptibility of *E. coli* (100%) to all carbapenems tested—ertapenem, imipenem, and meropenem—and nearly complete susceptibility to fosfomycin (99.71%) and nitrofurantoin (96.01%). These findings are in agreement with the study conducted by Duicu et al., which recommended these antibiotics for the empirical treatment of febrile or complicated UTIs in children [13]. Additionally, the lowest susceptibility in our study was seen for ampicillin, with a resistance rate of 62.28%. Moderate resistance rates were also observed for norfloxacin and trimethoprim–sulfamethoxazole. High susceptibility rates were maintained for ciprofloxacin (85.43%) and amoxicillin + clavulanic acid (75.05%), similarly to some global reported trends [45].

Our results are consistent with those of Rodríguez-Lozano et al. (2018) [53], who reported lower susceptibility rates to ampicillin and higher rates to ciprofloxacin and amoxicillin–clavulanic acid in their study on predominant uropathogens in pediatric urinary tract infections conducted in Spain between 2011 and 2015. Our findings suggest a generally favorable local susceptibility pattern, particularly with respect to carbapenems and fosfomycin, although caution is warranted due to notable resistance to ampicillin and moderate resistance to other agents. These patterns partially align with global trends in antimicrobial resistance, where *E. coli* has shown increasing resistance to first-line antibiotics. For instance, Pierantoni et al. in Italy observed growing resistance to amoxicillin–clavulanate (40.2%) and ciprofloxacin (16.3%), which differs from our higher susceptibility rates for these agents [54]. Similarly, Altamimi et al. (2023) in Saudi Arabia reported the presence of multidrug-resistant strains, though their findings also demonstrated high susceptibility to multiple antibiotic classes, including beta-lactams [55]. Shkalim Zemer et al. reported an increase in ESBL-producing *E. coli* isolates and corresponding resistance to amoxicillin + clavulanic acid and trimethoprim–sulfamethoxazole, findings that are partially reflected in our observed moderate resistance to trimethoprim–sulfamethoxazole [56]. Mahajan et al. in the United States reported increasing resistance to common antibiotics like amoxicillin and trimethoprim–sulfamethoxazole, trends that are also echoed to a degree in our results [46].

Our longitudinal analysis indicates a general trend toward the increased susceptibility of *Escherichia coli* to most β-lactam agents and amikacin between 2016 and 2022, while fluoroquinolone susceptibility remained relatively stable and norfloxacin demonstrated a marked decline. Ampicillin susceptibility increased modestly from approximately 30% to 42% over the study period; however, resistance remains prevalent in nearly two-thirds of isolates. This partial recovery contrasts with findings from a nationwide Israeli survey, which reported resistance rates consistently above 55% throughout 2007–2021 [56]. Susceptibility to amoxicillin–clavulanate remained relatively stable in the low 70% range but increased to approximately 81% by 2021–2022. This contrasts with outpatient surveillance in Portugal, where susceptibility declined from 94% to 65% over two decades [57], and the results reported from an Italian hospital cohorts—where the overall resistance among pediatric uropathogens was reported in nearly half of the isolates, though specific resistance to amoxicillin–clavulanate was not consistently quantified [58]. Among third- and fourth-generation cephalosporins, cefotaxime susceptibility improved significantly from 78% to 94%, and cefepime improved from 82% to 91%. These findings diverge from U.S. national trends showing a two- to threefold rise in third-generation cephalosporin resistance between 1999 and 2011 [59], but they are consistent with Italian data, where cefotaxime susceptibility remained high—above 90%—throughout 2012–2020, despite concerns about extended-spectrum β-lactamase (ESBL) production [58].

Improved *E. coli* susceptibility from 2016 to 2022 may likely reflect Romania’s national stewardship measures. In late 2016, the Ministry of Health launched the “No Antibiotics at Random” campaign to curb self-medication [60]. In the same year, Order 1101/2016 required every healthcare facility to establish an annual infection control and surveillance programme [60]. Government Decision 879/2018 then created the National Committee for Limiting Antimicrobial Resistance, introducing nationwide surveillance, retail sales tracking, and quarterly county feedback [61]. Further tightening followed with Order 487/2020, which discouraged antibiotics for mild COVID-19, and Law 3/2021, which made stewardship legally enforceable and introduced penalties for non-compliance [62,63].

Targeted stewardship programmes have also lowered resistance in *Enterobacteriaceae.* In Spain, a primary-care education and feedback programme reduced community infections caused by ESBL-producing *E. coli* by 66% within four years [64]. In a Korean children’s hospital, swapping third-generation cephalosporins for piperacillin–tazobactam cut ESBL rates in *E. coli* and *K. pneumoniae* from 42% to 17% [65]. An intensive-care unit in Taiwan linked a 60% drop in third-generation cephalosporins use to a marked fall in the ESBL isolates of both species [66]. At the community level, a Thai awareness campaign lowered the fecal carriage of ESBL-*Enterobacteriaceae* from 67% to 45% [67].

Several mechanistic studies explain how such gains occur. Experimental work shows that ESBL plasmids with measurable fitness costs are gradually lost when β-lactam pressure is removed [68]. Some plasmids impose little cost and can persist despite stewardship, highlighting the need for sustained pressure reduction and additional control measures [69]. Lastly, this analysis only includes community-acquired *E. coli* isolates; no hospital-acquired strains were analyzed. Community strains typically show lower and more stable resistance than hospital isolates, which are exposed to greater antibiotic pressure and harbor more ESBL and multidrug-resistant lineages [70,71]. Consequently, their susceptibility patterns and evolutionary trajectories may differ, and our findings should be interpreted within this outpatient context. This distinction is important because *E. coli* collected in hospital settings evolve under stronger antibiotic pressure and often follow different genetic trajectories, frequently acquiring additional resistance genes and integrons, than their community counterparts [72].

Our *Proteus mirabilis* findings align with pediatric data from the Mediterranean and Asia–Pacific regions [73,74]. In southeastern Turkey, Samanci and Pınarbaşı (2023) [75] recorded ampicillin susceptibility below 20%, together with persistently high activity of amikacin and meropenem—results that mirror our own. Portuguese surveillance showed amoxicillin–clavulanate resistance rising from 1.6% to 11.1%, close to the 12.8% observed in our series [57]. Carbapenems remain highly effective locally (96% susceptible to ertapenem and 94% to meropenem), matching the near-universal activity reported in Riyadh during the COVID-19 era [55]. Whereas a Taiwanese nationwide study documented ciprofloxacin susceptibility falling from 80% in 2002 to 54% in 2012 [76], we still observe 85% susceptibility. The apparently low imipenem activity in our cohort (23% susceptible, 48% intermediate) is probably a consequence of the 2023 CLSI/EUCAST breakpoint revision rather than emergent carbapenemases, an effect also noted among United States outpatients [77]. Complete resistance to nitrofurantoin confirms the intrinsic non-susceptibility of *Proteus mirabilis* first described by Stock in 2003 [78], and the continued efficacy of aminoglycosides is in accordance with data from Turkey [73].

The *Klebsiella pneumoniae* isolates included in our study displayed mixed yet largely encouraging susceptibility profiles. Complete (100%) resistance to ampicillin confirms the intrinsic non-susceptibility of *Klebsiella pneumoniae* and matches findings from southeast Turkey, where Samanci et al. reported >80% resistance in children [75]. By contrast, we observed 62.5% susceptibility to amoxicillin–clavulanate—noticeably higher than the around 45% reported in Portugal, Kuwait, and Italy, where resistance has risen since 2015 [57,79,80]. For piperacillin–tazobactam, two-thirds of our strains remained susceptible (62.5%), lower than the >80% activity recorded in Kuwaiti children but close to the 60–70% range noted in Faro, Portugal [57,79]. Third-generation cephalosporins retained good activity in Western Romania (73% susceptible to cefotaxime and ceftazidime), with similar rates being documented in Faro (72–75%), whereas Turkey reported higher resistance (cefuroxime resistance 46.7%) [74]. Carbapenems remained highly effective locally (95–100% susceptibility to ertapenem, imipenem, and meropenem), paralleling the near-complete activity reported in Kuwait and Turkey and underlining these agents as fallback options when ESBL production is suspected [75,79]. Aminoglycosides also performed well: 94.9% of our isolates were amikacin-susceptible, and 100% were gentamicin-susceptible, mirroring the >95% activity recorded in both Portugal and Kuwait [57,79]. Fluoroquinolone susceptibility showed early erosion: Only 57.5% of our isolates remained susceptible to ciprofloxacin, a lower rate than the ~80% still reported in Portugal but consistent with the rising resistance trend highlighted in Italian infants after 2015 [57,80]. Norfloxacin fared worse (39% susceptible), echoing Turkish observations of marked fluoroquinolone resistance among non-*E. coli* pathogens [75]. The apparent rise in norfloxacin resistance among *E. coli* isolates in the later years of the study is largely attributable to the updated EUCAST breakpoint criteria, which reclassified borderline inhibition zones as resistant, rather than reflecting true microbiological resistance [16]. Our fosfomycin susceptibility (45%) was lower than the >95% activity documented against ESBL-positive pediatric isolates in Seoul and the low (<10%) resistance reported in Portugal, suggesting caution when considering this oral option [57,81]. Nitrofurantoin proved the least reliable (25.7% susceptible), closely matching the one-third susceptibility described for Korean ESBL-positive *K. pneumoniae* and reflecting the rising nitrofurantoin resistance (47.5%) observed in Faro [57,81]. Finally, our trimethoprim–sulfamethoxazole susceptibility (62.5%) surpasses the poor activity reported in Turkey and Kuwait, where resistance exceeded 55%, yet it underscores that empirical use should be guided by rapid local surveillance given its variable efficacy worldwide [75,79].

Recent pediatric surveys confirm that *Enterococcus faecalis* susceptibility differs markedly between regions, underscoring the value of local data when selecting therapy [82,83,84]. In our community cohort, every isolate was ampicillin-susceptible, exceeding the 100% susceptibility reported from Crete [82] and the 93% seen in Tehran children’s hospitals [83] and sitting just above the global pooled estimate of 90% [84]. High-level gentamicin resistance affected 20% of our strains, lower than the 23% documented in Greek outpatients [82] and far below the 49% HLGR prevalence among Iranian pediatric in-patients [85]; it is also slightly better than the worldwide midpoint of 49.7% resistance We found that glycopeptides remained highly effective: 94% of our isolates were vancomycin-susceptible, closely matching the 100% reported in Crete, the 91% observed in Tehran, and the 96% global estimate [82,84,85]. Linezolid retained near-universal activity both locally and internationally, with around 1% resistance in the recent meta-analysis, confirming its continued role as a reserve agent [84]. Fluoroquinolone activity varied: Ciprofloxacin susceptibility reached 86% in our isolates, well above the 56% pooled global rate and dramatically higher than the 20% observed in the Iranian children’s hospital, illustrating substantial geographical heterogeneity [84,85]. Finally, although imipenem is not the first-line treatment for enterococci, its 59% in vitro activity here still surpasses the rising imipenem resistance clusters noted in the latest worldwide synthesis [86].

This study has several limitations that should be acknowledged. First, there was a higher number of children aged 1–5 years compared to other age groups, which may have influenced age-related estimates. Second, the study sample included a higher number of urban residents, potentially limiting the generalizability of findings across geographic settings. Third, we did not assess the monthly or seasonal differences in the UTI prevalence within the studied group. Fourth, we did not include questionnaires that assess the signs and symptoms, follow-up data, or risk factors of participants. Fifth, Bayesian analysis was not performed due to the lack of pre-existing regional data, which could have provided more refined insights by integrating prior knowledge with current observations. Finally, our data reflects the number and proportion of positive UTI samples (defined by the presence of leukocyturia and monomorphic bacterial growth) among the children tested rather than the true prevalence of UTIs in the general pediatric population. Because we did not have information on symptoms or the total number of children at risk, these results cannot be generalized to estimate population prevalence.

## 4. Materials and Methods

### 4.1. Study Design

We designed a retrospective observational study performed on consecutive children (aged between 1 and 18 years) from Arad County, Western Romania, who presented for routine testing at the County Emergency Clinical Hospital and Bioclinica Clinical Laboratories between 1 January 2016 and 31 December 2022. The Emergency Clinical Hospital stands as the largest healthcare facility in Arad County, Western Romania. Bioclinica Clinical Laboratories has several collection points in both the rural and urban areas of Arad County.

The inclusion criteria were as follows: children aged from 1 to 18 years; children with a legal guardian who agreed to participate; and children without serious or chronic diseases. The child’s disease status was verbally obtained from the primary caregiver by specialized nurses under the supervision of the primary researchers, without the use of written questionnaires.

Exclusion criteria included adult patients and infants aged below 1 year; children whose parents or legal guardian did not consent to the use of the child’s completely anonymized data for scientific research; samples collected from children with inconclusive test results as polymorphic flora between 10^5^ and 10^6^ colony-forming units (CFUs)/milliliter (mL); and samples collected from children with monomorphic flora above 10^6^ CFU/mL without significant leukocyturia.

### 4.2. Sample Collection

For younger children, urine was collected by the primary caregiver using non-invasive techniques, such as waiting for the child to void on their own before using a urine bag, pad, or clean catch of the urine stream [83,87]. If children were able, they were instructed to perform self-collection with the clean-catch midstream method. One urine sample per child was included in this survey. Urine test results were solely those obtained from children who were eligible for the study.

### 4.3. Culture

**Figure 1:**
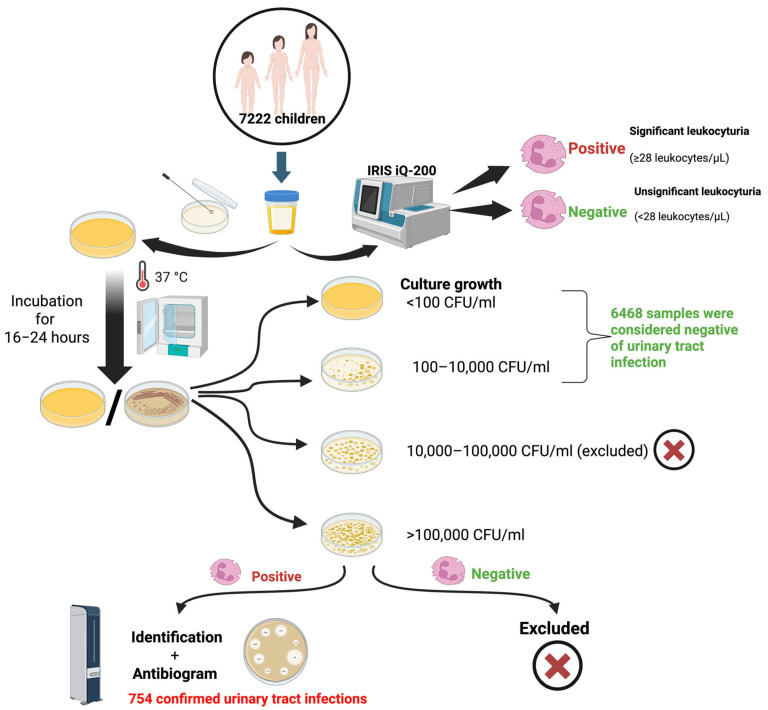
Screening and diagnosis algorithm to assess the presence of bacteria causing urinary tract infections in children from Western Romania. CFU, Colony-forming units; μL, microliter; mL, milliliter. Created in BioRender. Hermenean, A. (2025) https://BioRender.com/gy6yu7m.

All collected urine samples were inoculated onto CHROMID^®^ CPS^®^ ELITE (bioMérieux, Marcy-l’Étoile, France) culture media using a semi-quantified calibrated loop/surface streak method. The cultures were then incubated for 16 to 24 h at 37 °C [86] (Figure 1).

### 4.4. Interpretation of Results and Diagnosis Procedure

The following criteria were used to assess the colony count after incubation: I. Bacterial growth below 10,000 CFU/mL without the presence of leukocytes was considered to be negative for UTI; II. bacterial growth between 10,000 and 100,000 CFU with polymorphic flora and with/without leukocytes present was excluded from the study; III. bacterial growth >100,000 CFU/mL with monomorphic flora, as well as present leukocytes, was considered indicative of a UTI. For samples considered to be indicative of UTIs, bacterial identification and antibiogram were further performed [86] (Figure 1).

### 4.5. Assays

Urine microscopy examinations were conducted on all collected samples utilizing an automated urine microscopy analyzer: IRIS-iQ200 Series Analyzer (Beckman Coulter, Brea, CA, USA). Significant leukocyturia was considered to be >28 leukocytes/µL, in accordance with the manufacturer’s instructions.

For the identification and antimicrobial susceptibility testing of *Enterobacteriaceae* isolates, we utilized the Vitek^®^ 2 Compact automated system (BioMérieux, Inc., Hazelwood, MO, USA). Species identification was performed using appropriate Vitek ID cards, and when the identification confidence level was ≥90%—classified as “very good” or “excellent”—we proceeded with antibiotic susceptibility testing. For this, we employed the AST-N204 card (BioMérieux, Inc., Hazelwood, MO, USA), which includes a broad spectrum of antibiotics commonly used for Gram-negative pathogens. Antimicrobial susceptibility for *Enterococcus* spp. was determined with the Vitek 2 AST-592 card (BioMérieux, Inc., Hazelwood, MO, USA). The results of the antibiogram were interpreted as susceptible (S), intermediate (I), or resistant (R) in accordance with the European Committee on Antimicrobial Susceptibility Testing (EUCAST) guidelines and phenotypic interpretations valid for 2023 [16,86]. If the Vitek system failed to provide a definitive identification or if there was a clinical or microbiological suspicion, additional identification was attempted using matrix-assisted laser desorption/ionization time-of-flight mass spectrometry (MALDI-TOF).

### 4.6. Data Collection and Statistical Analyses

Data were completely anonymized so that any variable included in the study could not be attributed to a specific participant. The information collected for the database was age at the moment of urine collection (years), area of residence (rural/urban), gender (male or female), leukocytes present in the urine analysis (<28/µL or ≥28/µL), identified bacteria, and antibiogram sensitivity.

The data obtained was stored using Microsoft Excel, version 2011 (Microsoft Corp., Redmond, WA, USA). Statistical analyses were performed using Stata 16.1 (StataCorp, College Station, TX, USA). Data were presented as numbers, percentages, and mean ± standard deviation (SD).

Univariate logistic regression was used as the primary statistical model for this study. The crude odds ratios (cORs) with corresponding 95% confidence intervals (95% CIs) were reported for each variable. All variables were subsequently included in the multivariate logistic regression model to assess their independent associations, regardless of their significance in the univariate analysis. Adjusted odds ratios (aOR) with 95% CI were presented for the multivariate model.

Multinomial logistic regression was applied to evaluate year-to-year shifts in *E. coli* susceptibility for each antimicrobial. The results are expressed as relative risk ratios (RRRs) with 95% Cis and reported separately for two contrasts: susceptible versus intermediate (susceptible vs. intermediate) and susceptible versus resistant (susceptible vs. resistant). This approach captures both modest minimum inhibitory concentration drifts (reflected in the susceptible vs. intermediate comparison) and clinically relevant gains or losses in full resistance (susceptible vs. resistant), providing a nuanced view of temporal trends in antibiotic response. A *p*-value of <0.05 was considered statistically significant for univariate, multivariate, and multinomial analyses.

### 4.7. Ethical Approval

This study was approved by the Ethics Committee of “Vasile Goldis” Western University of Arad, Romania (no. 30 from 5 June 2024) and the Ethics Committee of Arad County Emergency Clinical Hospital (no. 63 from 26 March 2024).

## 5. Conclusions

Our study identified 10.44% of positive UTI samples in the investigated pediatric population, with *E. coli* as the predominant uropathogen. No significant difference in the number of positive UTI samples was found between urban and rural children. However, the results of the present survey suggest that UTI may be more frequently diagnosed in females compared to males. Overall, *E. coli* isolates demonstrated a high degree of susceptibility, particularly to carbapenems and fosfomycin. These results highlight the need for ongoing antimicrobial resistance surveillance and targeted educational efforts focused on early detection and prevention, particularly in higher-risk female pediatric populations. This study provides valuable epidemiological and antimicrobial resistance data that can aid pediatricians, general practitioners, microbiologists, and public health authorities in guiding empirical treatment decisions and shaping infection control policies. Future research should assess the risk factors using targeted questionnaires extended to other regions of Romania, regularly updating resistance profiles to ensure that treatment and prevention strategies remain tailored to local conditions.

## Figures and Tables

**Table 1:** Number of positive UTI samples by age, gender, and residence area in 7222 children from Western Romania.

Variable (*n*)	Samples Investigated for UTI		Univariate Logistic Regression		
Age Groups (Years)	Positive (%)	Negative (%)	cOR	95% CI	*p* Value
1–5 (3784)	336 (8.88)	3448 (91.12)		Ref.	
6–12 (2302)	276 (11.99)	2026 (88.01)	1.4	1.18–1.65	<0.001
13–18 (1136)	142 (12.5)	994 (87.5)	1.47	1.19–1.81	<0.001
**Gender**					
Male (2548)	143 (5.63)	2395 (94.37)		Ref.	
Female (4684)	611 (13.04)	4073 (86.96)	2.51	2.08–3.03	<0.001
**Area of residence**					
Rural (1667)	181 (10.86)	1486 (89.14)		Ref.	
Urban (5555)	573 (10.32)	4982 (89.68)	0.94	0.79–1.13	0.53
**Total** (7222)	754 (10.44)	6468 (89.56)			

UTI, Urinary tract infection; *n*, number of children; cOR, crude odds ratio; CI, confidence interval; Ref, reference.

**Table 2:** Yearly number of positive UTI samples in 7222 children from Western Romania (2016–2022).

	Samples Investigated for UTI		Univariate Logistic Regression		
Year (*n*)	Positive (%)	Negative (%)	cOR	95% CI	*p* Value
2016 (991)	123 (12.41)	868 (87.59)		Ref.	
2017 (908)	92 (10.13)	816 (89.87)	0.8	0.6–1.06	0.12
2018 (1121)	111 (9.9)	1010 (90.1)	0.78	0.59–1.02	0.07
2019 (1212)	124 (10.23)	1088 (89.77)	0.8	0.62–1.10	0.11
2020 (963)	102 (10.59)	861 (89.41)	0.84	0.63–1.10	0.21
2021 (1146)	115 (10.03)	1031 (89.97)	0.79	0.60–1.03	0.08
2022 (881)	87 (9.88)	794 (90.12)	0.77	0.58–1.03	0.08
**Total** (7222)	754 (10.44)	6468 (89.56)			

UTI, Urinary tract infection; *n*, total; cOR, crude odds ratio; CI, confidence interval; Ref., reference.

**Table 3:** Assessment of variables associated with the number of positive UTI samples in children from Western Romania.

Variable (*n*)	Samples Investigated for UTI		Multivariate Logistic Regression		
Age Groups (Years) (Ref.: 1–5)	Positive (%)	Negative (%)	aOR	95% CI	*p* Value
6–12 (2302)	276 (11.99)	2026 (88.01)	1.35	1.14–1.60	<0.001
13–18 (1136)	142 (12.5)	994 (87.5)	1.34	1.08–1.65	0.007
**Gender (Ref.: Males)**					
Females (4684)	611 (13.04)	4073 (86.96)	2.45	2.03–2.97	<0.001
**Area of Residence (Ref.: Rural)**					
Urban (5555)	573 (10.32)	4982 (89.68)	0.98	0.82–1.17	0.81
**Year (Ref.: 2016)**					
2017 (908)	92 (10.13)	816 (89.87)	0.82	0.62–1.10	0.19
2018 (1121)	111 (9.9)	1010 (90.1)	0.81	0.61–1.06	0.13
2019 (1212)	124 (10.23)	1088 (89.77)	0.84	0.64–1.09	0.19
2020 (963)	102 (10.59)	861 (89.41)	0.9	0.68–1.20	0.48
2021 (1146)	115 (10.03)	1031 (89.97)	0.84	0.64–1.10	0.2
2022 (881)	87 (9.88)	794 (90.12)	0.81	0.60–1.08	0.16

UTI, Urinary tract infection; *n*, total; aOR, adjusted odds ratio; CI, confidence interval; Ref., reference.

**Table 4:** Distribution of pathogens in pediatric UTI cases (*n* = 754) from Western Romania.

Pathogen	Number of Positive Urine Samples 754 (100%)
*E. coli*	501 (66.45%)
*Proteus mirabilis*	95 (12.6%)
*Klebsiella pneumoniae*	40 (5.31%)
*Enterococcus faecalis*	35 (4.64%)
*Pseudomonas aeruginosa*	19 (2.52%)
*Staphylococcus saprophyticus*	9 (1.19%)
*Streptococcus agalactiae*	9 (1.19%)
*Klebsiella oxytoca*	6 (0.8%)
*Coagulase negative Staphylococcus*	5 (0.66%)
*Enterococcus* spp.	4 (0.53%)
*Morganella morganii*	4 (0.53%)
*Enterobacter cloacae*	3 (0.4%)
Others *	24 (3.2%)

* Others include *Citrobacter freundii* (2/24), *Citrobacter koseri* (2/24), *Enterococcus faecium* (2/24), *Haemophilus influenzae* (2/24), *Proteus vulgaris* (2/24), *Salmonella* spp. (2/24), *Serratia marcescens* (2/24), *Streptococcus pyogenes* (2/24), *Citrobacter amalonaticus* (1/24), *Klebsiella* spp. (1/24), *Neisseria mucosa* (1/24), *Raoultella ornithinolytica* (1/24), *Sphingomonas paucimobilis* (1/24), *Staphylococcus aureus* (1/24), *Staphylococcus epidermidis* (1/24), and *Staphylococcus haemolyticus* (1/24).

**Table 5:** Antibiogram test results of *E. coli* isolates.

*E. coli*	Antibiotic Resistance		
Antibacterials (*n*)	Susceptible *n* (%)	Intermediate *n* (%)	Resistant *n* (%)
Ampicillin (501)	185 (36.93)	4 (0.8)	312 (62.28)
Amoxicillin + clavulanic acid (501)	376 (75.05)	56 (11.18)	69 (13.77)
Piperacillin + tazobactam (499)	434 (86.97)	26 (5.21)	39 (7.82)
Cefotaxime (478)	421 (88.08)	17 (3.56)	40 (8.37)
Ceftazidime (501)	437 (87.23)	32 (6.39)	32 (6.39)
Cefepime (451)	403 (89.36)	21 (4.66)	27 (5.99)
Ertapenem (451)	451 (100)	0	0
Imipenem (501)	501 (100)	0	0
Meropenem (501)	501 (100)	0	0
Amikacin (451)	391 (86.7)	59 (13.08)	1 (0.22)
Gentamicin (501)	454 (90.62)	1 (0.2)	46 (9.18)
Ciprofloxacin (501)	428 (85.43)	8 (1.6)	65 (12.97)
Norfloxacin (334)	185 (55.39)	0 (0)	149 (44.61)
Fosfomycin (350)	349 (99.71)	0 (0)	1 (0.29)
Nitrofurantoin (501)	481 (96.01)	17 (3.39)	3 (0.6)
Trimethoprim + sulfamethoxazole (501)	318 (63.47)	0	183 (36.53)

*n*, Total strains tested.

**Table 6:** Longitudinal multinomial analysis of community-acquired *E. coli* antibiotic susceptibility trends, 2016–2022.

*E. coli*		Antibiotic Resistance			Multinominal Statistical Analysis		
Antibacterials (*n*)	Year	Susceptible	Intermediate	Resistant	RRR	95% CI	*p* Value
	**(*n* = 100%)**	***n* (%)**	***n* (%)**	***n* (%)**			
Ampicillin (501)	2016 (77)	23 (29.87)	0	54 (70.13)			
	2017 (60)	21 (35)	1 (1.67)	38 (63.33)			
	2018 (83)	24 (28.92)	0	59 (71.08)	1.02 *	0.61–1.71 *	0.93 *
	2019 (79)	32 (40.51)	1 (1.27)	46 (58.23)	0.9 **	0.82–0.99 **	0.025 **
	2020 (72)	29 (40.28)	1 (1.39)	42 (58.33)			
	2021 (73)	32 (43.84)	1 (1.37)	40 (54.79)			
	2022 (57)	24 (42.11)	0	33 (57.89)			
Amoxicillin + clavulanic acid (501)	2016 (77)	54 (70.13)	10 (12.99)	13 (16.88)			
	2017 (60)	46 (76.67)	10 (16.67)	4 (6.67)			
	2018 (83)	60 (72.29)	13 (15.66)	10 (12.05)	0.8 *	0.68–0.93 *	0.003 *
	2019 (79)	58 (73.42)	10 (12.66)	11 (13.92)	1.03 **	0.9–1.17 **	0.7 **
	2020 (72)	52 (72.22)	10 (13.89)	10 (13.89)			
	2021 (73)	60 (82.19)	3 (4.11)	10 (13.7)			
	2022 (57)	46 (80.7)	0	11 (19.3)			
Piperacillin + tazobactam (499)	2016 (77)	64 (83.12)	9 (11.69)	4 (5.19)			
	2017 (60)	52 (86.67)	6 (10)	2 (3.33)			
	2018 (83)	71 (85.54)	4 (4.82)	8 (9.64)	0.69 *	0.55–0.88 *	0.002 *
	2019 (78)	68 (87.18)	1 (1.28)	9 (11.54)	1.07 **	0.9–1.27 **	0.43 **
	2020 (72)	64 (88.89)	3 (4.17)	5 (6.94)			
	2021 (72)	63 (87.5)	3 (4.17)	6 (8.33)			
	2022 (57)	52 (91.23)	0	5 (8.77)			
Cefotaxim (478)	2016 (77)	60 (77.92)	9 (11.69)	8 (10.39)			
	2017 (60)	53 (88.33)	4 (6.67)	3 (5)			
	2018 (83)	71 (85.54)	4 (4.82)	8 (9.64)	0.4 *	0.26–0.64 *	<0.001 *
	2019 (79)	73 (92.41)	0	6 (7.59)	0.92 **	0.77–1.1 **	0.35 **
	2020 (72)	61 (84.72)	0	11 (15.28)			
	2021 (73)	71 (97.26)	0	2 (2.74)			
	2022 (34)	32 (94.12)	0	2 (5.88)			
Ceftazidim (501)	2016 (77)	60 (77.92)	13 (16.88)	4 (5.19)			
	2017 (60)	53 (88.33)	5 (8.33)	2 (3.33)			
	2018 (83)	70 (84.34)	6 (7.23)	7 (8.43)	0.74 *	0.6–0.9 *	0.003 *
	2019 (79)	73 (92.41)	0	6 (7.59)	0.94 **	0.78–1.14 **	0.55 **
	2020 (72)	61 (84.72)	0	11 (15.28)			
	2021 (73)	68 (93.15)	4 (5.48)	1 (1.37)			
	2022 (57)	52 (91.23)	4 (7.02)	1 (1.75)			
Cefepim (451)	2016 (44)	36 (81.82)	8 (18.18)	0			
	2017 (45)	39 (86.67)	4 (8.89)	2 (4.44)			
	2018 (82)	72 (87.8)	4 (4.88)	6 (7.32)	0.67 *	0.51–0.87 *	0.003 *
	2019 (79)	73 (92.41)	0	6 (7.59)	0.99 **	0.8–1.23 **	0.95 **
	2020 (72)	61 (84.72)	0	11 (15.28)			
	2021 (73)	71 (97.26)	1 (1.37)	1 (1.37)			
	2022 (56)	51 (91.07)	4 (7.14)	1 (1.79)			
Amikacin (451)	2016 (44)	23 (52.27)	21 (47.73)	0			
	2017 (45)	28 (62.22)	17 (37.78)	0			
	2018 (82)	72 (87.8)	10 (12.2)	0			
	2019 (79)	77 (97.47)	2 (2.53)	0	0.49 **	0.4–0.59 **	<0.001 **
	2020 (72)	68 (94.44)	4 (5.56%)	0			
	2021 (73)	70 (95.89)	3 (4.11%)	0			
	2022 (56)	53 (94.64)	2 (3.57%)	1 (1.79)			
Ciprofloxacin (501)	2016 (77)	61 (79.22)	0	16 (20.78)			
	2017 (60)	52 (86.67)	0	8 (13.33)			
	2018 (83)	76 (91.57)	0	7 (8.43)	2.31 *	1.25–4.25 *	0.008 *
	2019 (79)	68 (86.08)	0	11 (13.92)	0.9 **	0.79–1.04 **	0.15 **
	2020 (72)	60 (83.33)	2 (2.78)	10 (13.89)			
	2021 (73)	63 (86.3)	3 (4.11)	7 (9.59)			
	2022 (57)	48 (84.21)	3 (5.26)	6 (10.53)			
Norfloxacin *** (334)	2016 (77)	59 (76.62)	0	18 (23.38)			
	2017 (60)	44 (73.33)	0	16 (26.67)			
	2018 (83)	71 (85.54)	0	12 (14.46)	2.29 **	1.94–2.71 **	<0.001 **
	2019 (12)	11 (91.67)	0	1 (8.33)			
	2020 (0)	0	0	0			
	2021 (45)	0	0	45 (100)			
	2022 (57)	0	0	57 (100)			
Trimethoprim + sulfamethoxazole (501)	2016 (77)	39 (50.64)	0	38 (49.36)			
	2017 (60)	33 (55)	0	27 (45)			
	2018 (83)	60 (72.28)	0	23 (27.72)	0.9 **	0.82–0.99 **	0.027 **
	2019 (79)	52 (65.82)	0	27 (34.18)			
	2020 (72)	47 (65.28)	0	25 (34.72)			
	2021 (73)	47 (64.38)	0	26 (35.62)			
	2022 (57)	40 (70.18)	0	17 (29.82)			

*n* = Total; RRR: relative risk ratios; CI = confidence interval; * = susceptible compared with intermediate; ** = susceptible compared with resistant; *** = adoption of EUCAST fluoroquinolone breakpoints (v 12.0) in 2021 reclassified borderline isolates as resistant, accounting for the apparent loss of norfloxacin susceptibility [16].

**Table 7:** Antibiogram test results of *Proteus mirabilis* isolates.

*Proteus mirabilis*	Antibiotic Resistance		
Antibacterials (*n*)	Susceptible	Intermediate	Resistant
	*n* (%)	*n* (%)	*n* (%)
Ampicillin (93)	35 (37.64)	4 (4.3)	54 (58.06)
Amoxicillin + clavulanic acid (78)	61 (78.21)	7 (8.97)	10 (12.82)
Piperacillin + tazobactam (95)	83 (87.36)	6 (6.32)	6 (6.32)
Cefotaxime (87)	77 (88.5)	7 (8.05)	3 (3.45)
Ceftazidime (95)	82 (86.32)	5 (5.26)	8 (8.42)
Cefepime (85)	73 (85.88)	7 (8.24)	5 (5.88)
Ertapenem (81)	78 (96.3)	3 (3.7)	0
Imipenem (95)	22 (23.16)	46 (48.42)	27 (28.42)
Meropenem (95)	89 (93.68)	3 (3.16)	3 (3.16)
Amikacin (85)	76 (89.41)	9 (10.59)	0
Gentamicin (95)	80 (84.21)	1 (1.05)	14 (14.74)
Ciprofloxacin (95)	81 (85.27)	1 (1.05)	13 (13.68)
Norfloxacin (67)	42 (62.69)	0	25 (37.31)
Fosfomycin (42)	35 (83.33)	0	7 (16.67)
Nitrofurantoin (76)	0	0	76 (100)
Trimethoprim + sulfamethoxazole (95)	43 (45.26)	0	52 (54.74)

*n*, Total strains tested.

**Table 8:** Antibiogram test results of *Klebsiella pneumoniae* isolates.

*Klebsiella pneumoniae*	Antibiotic Resistance		
Antibacterials (*n*)	Susceptible	Intermediate	Resistant
	*n* (%)	*n* (%)	*n* (%)
Ampicillin (40)	0	0	40 (100)
Amoxicillin + clavulanic acid (40)	25 (62.5)	6 (15)	9 (22.5)
Piperacillin + tazobactam (40)	25 (62.5)	5 (12.5)	10 (25)
Cefotaxime (39)	29 (74.36)	3 (7.69)	7 (17.95)
Ceftazidime (40)	29 (72.5)	3 (7.5)	8 (20)
Cefepime (39)	29 (74.36)	4 (10.26)	6 (15.38)
Ertapenem (37)	37 (100)	0	0
Imipenem (40)	38 (95)	2 (5)	0
Meropenem (40)	38 (95)	2 (5)	0
Amikacin (39)	37 (94.87)	2 (5.13)	0
Gentamicin (40)	40 (100)	0	0
Ciprofloxacin (40)	23 (57.5)	1 (2.5)	16 (40)
Norfloxacin (23)	9 (39.13)	1 (4.35)	13 (56.52)
Fosfomycin (20)	9 (45)	0	11 (55)
Nitrofurantoin (35)	9 (25.71)	14 (40)	12 (34.29)
Trimethoprim + sulfamethoxazole (40)	25 (62.5)	0	15 (37.5)

*n*, Total strains tested.

**Table 9:** Antibiogram test results of *Enterococcus faecalis* isolates.

*Enterococcus faecalis*	Antibiotic Resistance		
Antibacterials (*n*)	Susceptible	Intermediate	Resistant
	*n* (%)	*n* (%)	*n* (%)
Ampicillin	35 (100)	0	0
Imipenem	10 (58.82)	7 (41.18)	0
High-Level Gentamicin	28 (80)	0	7 (20)
High-Level Streptomycin	22 (62.86)	0	13 (37.14)
Ciprofloxacin	30 (85.71)	3 (8.57)	2 (5.71)
Linezolid	32 (94.12)	2 (5.88)	0
Teicoplanin	33 (94.29)	0	2 (5.71)
Vancomycin	33 (94.29)	0	2 (5.71)
Tigecycline	35 (100)	0	0

*n*, Total strains tested.

## Data Availability

All relevant data are presented in the study.

## Abbreviations

The following abbreviations are used in this manuscript:

CFU	Colony-forming unit;
UTI	Urinary tract infection;
Spp.	Several species;
ESBL	Extended-spectrum beta-lactamase.
